# User-Centered Virtual Clinic Services for Remote, Rural, and Underserved Sub-Saharan Africa: Development and Usability Study

**DOI:** 10.2196/84272

**Published:** 2026-09-01

**Authors:** Abby Blocker, Ahmed Biyabani, Joyce Mwangama, Mohammed Ishaaq Datay, Bessie Malila

**Affiliations:** 1 Division of Biomedical Engineering, University of Cape Town, 7th Floor, Anatomy Building, 1 Anzio Road, Observatory, Cape Town, 7925, South Africa, 27 021 650 3093; 2 Carnegie Mellon University Africa, Kigali, Rwanda; 3 Department of Electrical Engineering, University of Cape Town, Cape Town, South Africa; 4 Primary Health Care Directorate, University of Cape Town, Cape Town, South Africa; 5 Department of Electrical and Electronic Engineering Science, University of Johannesburg, Johannesburg, South Africa

**Keywords:** telemedicine, remote consultation, digital health, user-centered design, South Africa, Africa South of the Sahara, rural health, resource-limited settings, medically underserved area, primary health care

## Abstract

**Background:**

Virtual clinics allow doctors to connect to patients in difficult-to-reach locations. While this can result in improved access to care, implementing existing virtual clinics in these locations is difficult due to their contextual constraints. To address these challenges, a virtual clinic system for remote, rural, and underserved areas was developed based on user-centered design principles. To complete the user-centered design cycle, the developed system was then implemented and evaluated in the target contexts.

**Objective:**

This study aimed to conduct a pilot study with the developed user-centered virtual clinic system to evaluate its performance in resource-limited settings. Patient, doctor, and nurse feedback was collected to understand how the virtual clinic system and implementation strategy might be improved before long-term implementation.

**Methods:**

A pilot study was conducted at 2 health care facilities in South Africa—1 underserved public primary health clinic in the city of Cape Town, and 1 remote occupational health clinic in the Northern Cape Province. Doctor and nurse dyads participated in using the system to consult with real patients in each clinic. Patients received both virtual and physical examinations by the same doctor, and these 2 consultations were compared with one another. Surveys were conducted to gather patient feedback pre– and post–virtual clinic consult. Observations of system usage were collected, and doctor and nurse participant interviews were conducted at the conclusion of each day. Qualitative data were thematically analyzed to understand barriers and facilitators of virtual consultations, as well as patient satisfaction.

**Results:**

A total of 12 patient consultations were conducted using the virtual clinic system across 2 health care facilities. Two doctors and 2 nurses participated in the study as users. Doctor and nurse confidence in using the system increased over time. Doctors were confident that the virtual consultation format could be used to diagnose patients remotely. Key indicators of patient satisfaction were being included in consultation communication and understanding the benefits virtual care could offer. Potential barriers to virtual consultations were infrastructure offered by the implementation environment and medical device limitations.

**Conclusions:**

The results from this pilot study indicate that virtual clinic consultation is possible in low-resource settings using the developed virtual clinic system. This system can support patients in remote, rural, and underserved areas to receive certain health care services from a doctor without the doctor having to be physically present in the facility. The results encourage further scaling of the system to support long-term implementation in low-resource health clinics in South Africa and other sub-Saharan African countries.

## Introduction

### Background

Virtual clinics are a type of digital health solution that connect health care professionals to patients and other staff through digital means [1]. Implementation of virtual clinics is associated with many advantages: better quality and continuity of care [2-5], greater efficiency leading to reduced wait times [6,7], and reduced costs for both patients and health systems [8,9]. Due to the COVID-19 pandemic, the number of virtual clinics has grown [10], and implementations have been realized globally with a range of different applications.

### Virtual Clinics in Sub-Saharan Africa

While virtual clinics have become common in many urban and developed areas, they have not yet reached many remote, rural, and underserved areas. This is because these areas present contextual challenges, including a lack of telecommunication infrastructure [11,12], limited access to electricity [13], and low levels of digital literacy [14]. Current virtual clinic services have not been adequately designed for these constraints, contributing to a lack of adoption in these areas. Furthermore, populations in remote, rural, and underserved areas often lack proper health facilities and skilled health professionals [15]. Virtual clinics have the potential to greatly improve access to care in these areas [16].

National programs in sub-Saharan Africa have attempted to implement digital health policy and services with mixed results. A national telemedicine program was implemented in Cabo Verde to connect doctors across the 9 inhabited islands of the country [16]. There were several technical challenges with this implementation, including a cyberattack that affected service provision, extensive financial investment, and the inability to maintain the hardware. Evidence collected in a review of telemedicine in sub-Saharan Africa corroborates these challenges [17]. Ogundaini and Mlitwa [18] discuss how digital health initiatives across sub-Saharan Africa require improved capacity building, knowledge sharing, and resources to be successful. South Africa, Kenya, and Nigeria have emerged as leaders on the continent for telemedicine implementations; however, digital literacy limitations, regulatory gaps, and infrastructure constraints were mentioned as limiting factors to success [19]. Implementation recommendations from the World Health Organization [16] and the International Telecommunication Union [20] provide some guidance for addressing these challenges, but alignment between recommendation and existing digital health system design still persists.

### User-Centered Design for Virtual Clinics

User-centered design (UCD) is a methodology that may be applied during the design and development process to align the needs of the context with the digital health system [21-25]. UCD focuses on user needs to iteratively develop solutions [26,27]. There are 4 steps in the UCD process, which are (1) specify context of use, (2) specify requirements, (3) create design solutions, and (4) evaluate designs. Incorporation of UCD can lead to improved user interfaces and functionalities within the system. By incorporating user feedback early in the design process, development time decreases and user acceptance increases [28,29]. Several virtual clinics that have implemented UCD methods have shown improved user experience and system uptake [30,31].

Previous work outlined the application of UCD methods to develop a virtual clinic system for sub-Saharan Africa [32]. In brief, a situational analysis was conducted to understand the challenges associated with health care provision and digital health in austere environments in sub-Saharan Africa and South Africa. This addressed step 1 of the UCD process. From this situational analysis, system requirements were developed, addressing UCD step 2. The virtual clinic was designed according to these requirements, completing UCD step 3. Initial usability testing was conducted with nurses and doctors in Cape Town, completing UCD step 4. This completed the initial UCD iteration. Another UCD iteration was then begun by using the feedback from the usability study to further improve the next prototype.

The virtual clinic was designed for provider-to-provider synchronous telemedicine. In this model, a patient would visit their regular health clinic in a remote, rural, or underserved location. It is assumed that there would be limited or no doctors available to provide services in person. If the nurse or community health worker is unsure how to diagnose or treat a patient, they would typically refer the patient to a district or tertiary health facility or ask them to wait for the doctor to visit the facility. Instead, using the virtual clinic system, the nurse can call an available doctor who can advise on how to proceed with the patient remotely. The patient would either be adequately treated via the virtual consultation, or the doctor can confirm that the patient should be referred. This reduces unnecessary referrals, which is an advantage for both the patient and the health care system.

This design acknowledges the needs of low-resource settings in several ways. First, the provider-to-provider model reduces the onus on the patient that is typically associated with virtual care. The patient does not need to purchase any devices or have proper infrastructure such as electricity or internet in their home to participate in virtual consultations. The nurse is the user who facilitates the digital connection for the patient. Additionally, the system and infrastructure can be concentrated at a central point (the existing clinic facility) and shared among many patients, addressing the potential lack of resources for health care in these areas. Finally, it allows patients to have access to a doctor. Many patients do not fulfill referral appointments for various reasons, including cost, lack of transportation, long distances with poor health, and lack of child or older adult care. By implementing the virtual clinic system, barriers to care are reduced, and patients have an opportunity to receive better quality health care.

### Research Objectives

In this paper, we present results of the evaluation of the virtual clinic system that was developed using UCD methods in an effort to address access to care in remote, rural, and underserved areas in sub-Saharan Africa. The improved system was evaluated during this study in real-world contexts, completing step 4 and thus the second iteration of the UCD process.

The UCD method emphasizes usability and contextually relevant design, so this evaluation was focused on understanding how the virtual clinic system performed in real-world environments and how the users experienced the system. Specifically, the objectives were to understand how the virtual clinic performed in a real-world setting and to identify potential barriers that could be encountered during a scaled-up implementation, which answer the following three research questions: (1) What barriers do users encounter when delivering primary-level care using the virtual clinic? (2) What user behaviors are commonly present in successful virtual clinic consultations? (3) How satisfied are patients with receiving primary-level care using the virtual clinic? The following sections present the methods used for answering these research questions, the results of the data collection, and the recommendations for future implementation, as well as the limitations of this study.

## Methods

### Theoretical Background

There is extensive literature documenting the evaluation of telemedicine systems, and these evaluations vary significantly in their objectives. Ekeland et al [33] provide a review of methodologies for evaluating telemedicine systems and highlight the difficulties in aligning these methodologies. They highlight that telemedicine exists at the intersection of medical, technological, and social sciences, and it can be difficult to reconcile evaluation methods that satisfy each of these fields. The differences can be summarized as positivism versus naturalism. Positivism uses objective observation and measurement to develop generalizable laws, while naturalism focuses on understanding phenomena within their natural context, often through qualitative methods, and acknowledges the influence of various factors on human behavior. Both approaches can yield valuable insights, with positivist evaluations such as randomized controlled trials being important for establishing clinical and generalized outcomes and naturalist evaluations establishing context-relevant information and human experience and behaviors. They highlight that the context of the telemedicine implementation and objectives of the evaluation should dictate the appropriate approach.

In regions where telemedicine is still evolving, such as sub-Saharan Africa and South Africa, the strict design of positivist evaluations may not always be appropriate. Randomized controlled trials may not account for rapid developments in technology, leading to results that may not be relevant by the time the study concludes [34]. Furthermore, in contexts where telemedicine has not yet been implemented, gathering human experience and feedback via a naturalistic approach can be advantageous to understand the cultural and contextual effects of telemedicine implementations [35]. This also allows for refinement of implementation techniques that are context-based.

Given that the developed virtual clinic system is still at an early technology readiness level, and telemedicine within remote, rural, and underserved areas in South Africa has not yet been widely attempted, the evaluation approach used in this study leans naturalist rather than positivist. However, elements of both are incorporated to strengthen the study design and outcomes. Therefore, a mixed methods feasibility study was used to evaluate the virtual clinic.

### Virtual Clinic System

**Figure 1.**
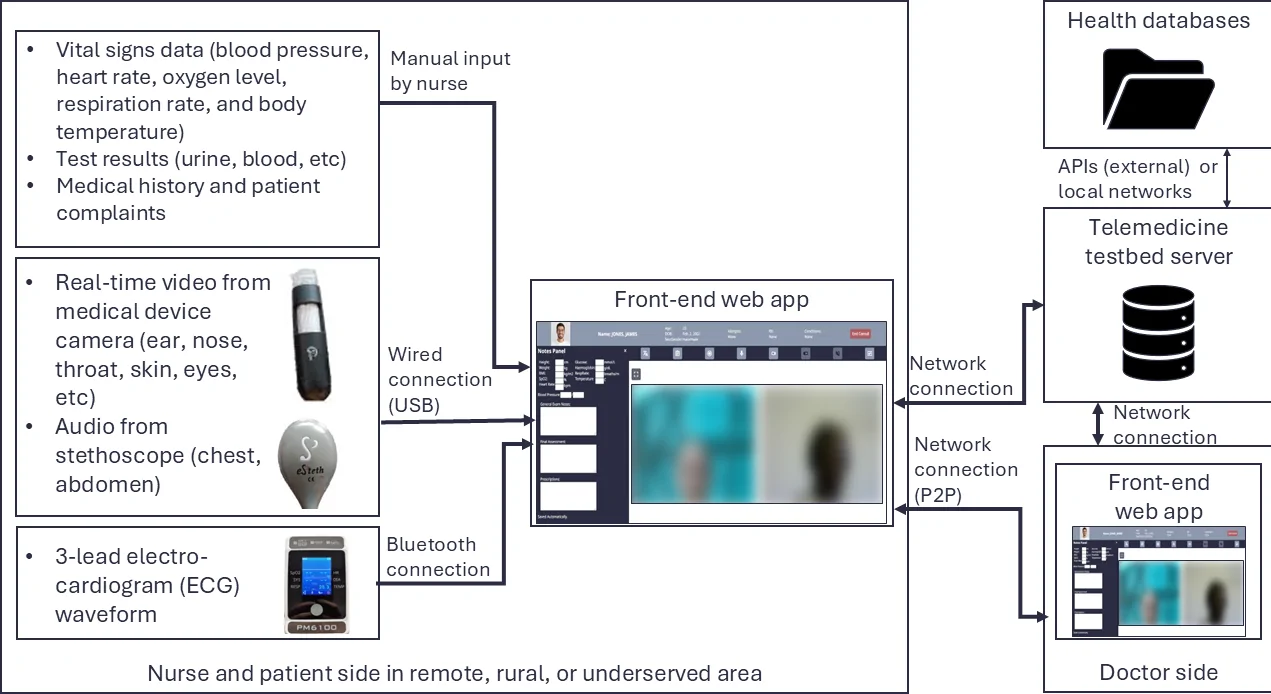
Virtual clinic platform architecture.

The developed virtual clinic system was used for virtual consultations during this study. The system consists of a web app with integrated medical devices, as described in Figure 1. It includes three main subsystems: (1) the consultation platform, which facilitates real-time communication and transmits real-time health data; (2) the electronic health record, which houses patient health data and consultation data from consultations hosted on the virtual clinic platform; and (3) a help desk to support user training and troubleshooting while using the system. Each of the subsystems was designed with user input and consideration of the contexts for which the system was intended.

There are three main medical devices that were integrated with the web app: (1) a digital stethoscope, which collects real-time auscultation sounds; (2) a medical camera, which provides general-purpose high-quality magnified video of the patient and has an otoscope adapter to provide ear, nose, and throat video; and finally (3) a vital signs monitor that can transmit 3-lead electrocardiogram readings via Bluetooth. The medical camera, which provides magnified medical images, is distinct from the webcam, which provides a real-time video of the nurse and patient interactions to the doctor. Images, recordings, and notes are captured during virtual consultations and stored in the electronic health record subsystem, so that this information can be referenced in future consultations. The devices used in this study can be substituted with similar devices. This allows clinics the ability to interchange devices depending on their budget, medical needs, and market availability in the region of implementation. For example, if the digital stethoscope used in the implementation in South Africa is not sold in other countries, implementers could replace it with a similar device, which may also be cheaper. This is in contrast to many existing telemedicine systems, in which medical devices and hardware are linked and cannot be interchanged. This design improves feasibility in the target contexts of remote, rural, and underserved areas.

A help desk is also integrated into the consultation platform. The help desk contains extensive training material for users, including video demonstrations and written guides on how to use the medical devices and software. There are also 4 case studies developed as training material, which users can go through as independent practice with the system. This setup allows users to become comfortable with the tools on their own time, rather than assuming that training time will be provided during implementation.

### Study Sites and Participant Recruitment

Two study sites were used for virtual clinic implementation: Mitchell’s Plain Community Health Centre in Cape Town and the Occupational Health Clinic at the Klerefontein Base for the South African Radio Astronomy Observatory (SARAO) Square Kilometre Array (SKA) project in Northern Cape Province. These sites are hereafter referred to as sites 1 and 2.

Site 1 is a public health facility in Mitchell’s Plain township in Cape Town. It is estimated that the clinic serves approximately 1.2-million patients, including Mitchell’s Plain and surrounding areas, with approximately 46,000 patients seen per month [36]. Despite being located in the urban center of Cape Town, there are still difficulties involved in getting doctors to the facility. Safety is a concern in Mitchell’s Plain; the area has the highest reported assault and sexual assault rates in South Africa and is in the top 10 areas for robbery-related crimes [37]. This can make it difficult for doctors to get in and out of the facility. Therefore, virtual clinics offer a potential solution for remote clinical consultation without requiring doctors to physically visit the clinic. The study was conducted over 2 consecutive weekend days at site 1.

Site 2 is an occupational health clinic that serves approximately 200 employees at the SARAO SKA site. The nearest town is Carnarvon, which is approximately 16 km away. The clinic has an on-site nurse who provides day-to-day care. In addition, an occupational health nurse and doctor fly to the site once every 3 months for 1 day. During these visits, medical consultations are conducted for approximately 5 hours. The on-site nurse arranges for the patients who are in most need of the doctor to come to the clinic during these site visits. Alternatively, the workers may visit the community health clinic or the district hospital in Carnarvon, where there are an estimated 3 doctors who provide services between these sites. These facilities can also provide some trauma care in case of an emergency, although more serious cases of trauma must be transported to the tertiary hospital in De Aar, which is 200 km from Carnarvon. At site 2, the study was conducted over 1 day, during a site visit by the occupational health doctor.

**Figure 2.**
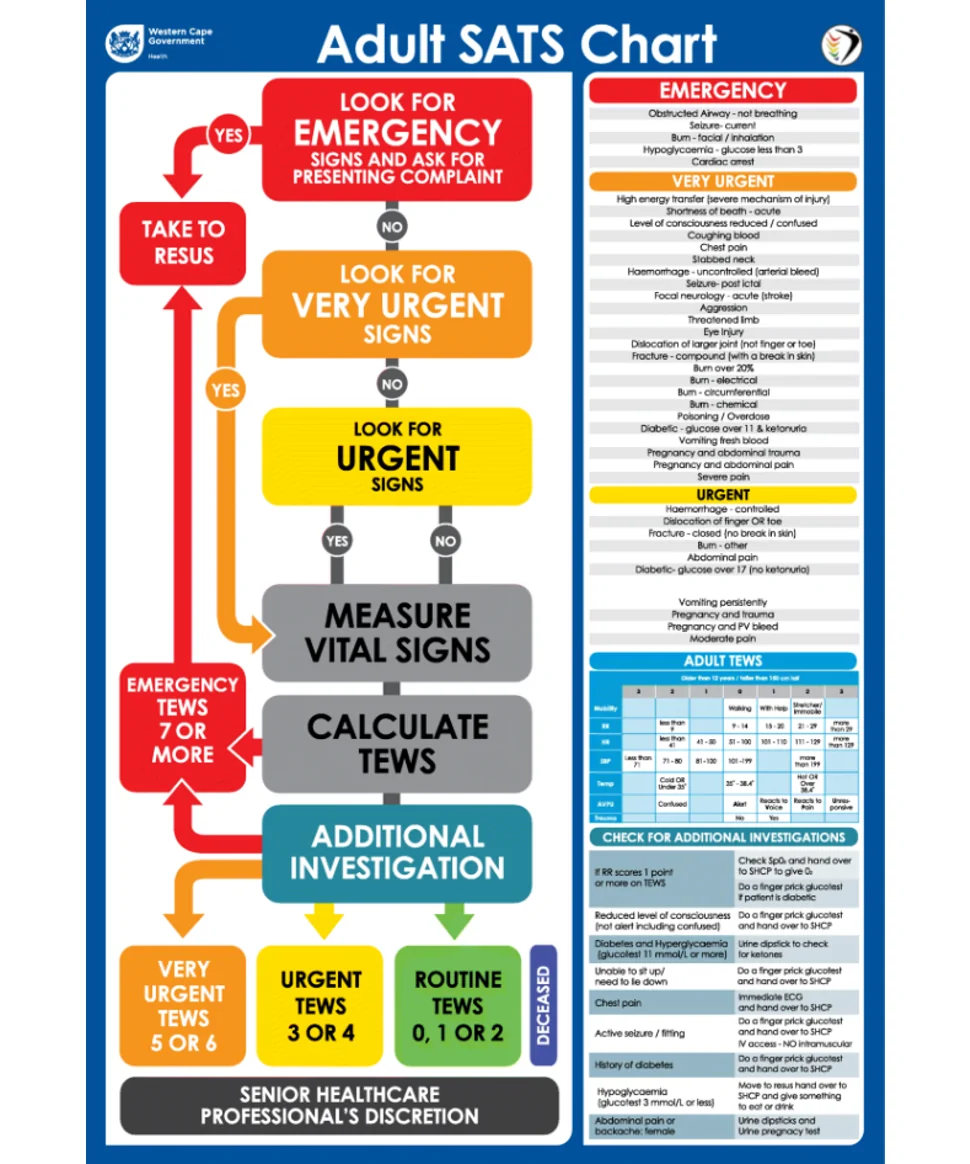
The SATS flowchart for adults (adapted from the SATS training manual) [34]. SATS: South African Triaging Scale.

Patients were recruited from each clinic using a convenience sampling method. Inclusion criteria for patients were adults who were fluent in English. Patients were also required to be triaged as level green according to the South African Triaging Scale protocol, shown in Figure 2 [38]. Level green is the lowest risk patient, with a routine-reported Triage Early Warning Score of <3. At site 1, patients underwent triaging after entering the clinic facility. At site 2, the on-site nurse recruited patients who fit the inclusion criteria in advance of the study day. These patients were scheduled for consultation times on the study day. Exclusion criteria were patient cases in which the main complaint required a procedure, such as a catheter replacement, as these cases did not involve a diagnosis and therefore did not fit the study design.

Doctors and nurses were recruited using a snowball sampling method. Participants recruited for the doctor role were required to have a valid Bachelor of Medicine and Bachelor of Surgery qualification and a current registration with the Health Professions Council of South Africa. Participants recruited for the nurse role were required to have a National Qualifications Framework level 7 nursing degree or diploma and current registration with the South African Nursing Council. The inclusion criteria for both doctors and nurses were that they should have no prior clinical experience using real-time virtual consultation systems.

### Study Design and Overview

**Figure 3.**
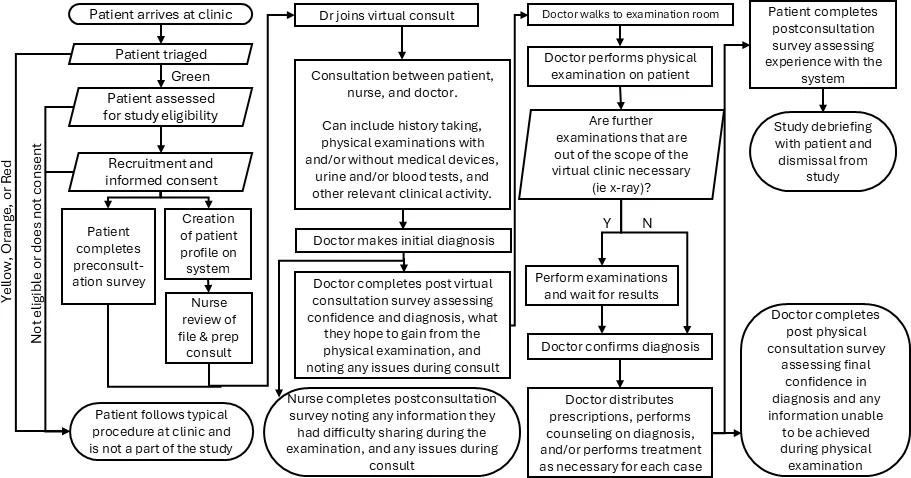
Study procedure used during the virtual clinic field pilot study.

To evaluate the developed virtual clinic system, a mixed methods, cross-sectional, pre-experimental pilot feasibility study was conducted. The virtual clinic system was implemented in 2 field clinic settings with patient, doctor, and nurse participants. A detailed depiction of the consultation study procedure is shown in Figure 3. The study commenced by training the doctor and nurse participants on the virtual clinic system. For doctors, the training took place the morning of their first study day for approximately 30 minutes. A dedicated training session was done for the nurses a day before the system evaluation at the clinic. This was because the nurses needed to familiarize themselves with the medical devices, whereas the doctors only viewed the data collected from the devices, which did not require extensive training. Consultation sessions began in the clinic after the doctor and nurse completed their training on the system. During the system evaluation phase, patients participated in a virtual consultation facilitated by the nurse, with a doctor present virtually. The doctor was located in the same health facility as the nurse and the patient, but in a separate room, so they could not hear or see the examination other than through the virtual clinic system. The nurse then facilitated the conversation between the doctor and the patient, provided the medical history, and performed physical examinations as instructed by the doctor. After the virtual examination was finished, the doctor then entered the examination room and performed a physical examination to compare virtual and physical examination results. The study day concluded by conducting dyadic interviews with the doctor and the nurse about their experience with the system.

### Data Collection and Analysis

Both quantitative and qualitative data were collected during the study. A total of 5 different structured, self-report questionnaires were administered, which included 2 patient questionnaires, 2 doctor questionnaires, and 1 nurse questionnaire. A mixture of quantitative (scale rankings or binary) and qualitative (open answer) questions was included. The questionnaires are provided in Multimedia Appendix 1. Clinical notes were recorded during each section by the doctors and nurses in the virtual clinic platform. Simple observational data were collected by the researcher, which included the duration of each consult, examinations performed, and challenges encountered. Dyadic unstructured interviews were conducted with the nurse and the doctor at the conclusion of each study day to gather self-reported user feedback regarding the virtual clinic system.

Regarding research question 2 (What user behaviors are commonly present in successful virtual clinic consultations?), a successful virtual clinic consultation was defined as a consultation conducted using the virtual clinic where the doctor came to a diagnosis during the virtual consultation and reported a confidence level equal to that reported during the physical consultation. Regarding question 3, patient satisfaction was defined as a combination of “quality of care, similarity to face-to-face encounter, perception of the interaction” [39], willingness to use the service again, and willingness to recommend the service. Two standardized tools were incorporated within the patient and doctor questionnaires: the Telehealth Satisfaction Survey (TeSS) and a 5-point Likert scale diagnostic confidence ranking, respectively. TeSS was used to understand patient satisfaction during virtual consults. This questionnaire was developed by Yip et al [39] and has since been used to evaluate rural patients’ satisfaction with telemedicine in several studies [40,41]. To define successful consultations, a 5-point Likert scale was used to assess doctor confidence in virtual and physical diagnosis. This method has previously been used to rank confidence in diagnosis across many domains [42,43]. Although more detailed surveys have been developed for assessing diagnostic confidence, such as the Primary Care Confidence Scale [44], due to the short time span between virtual and physical consultations it was important to use a quick and simple questionnaire. Thus, the Likert scale confidence ranking was used.

The data collected were transcribed and cleaned as necessary to remove identifying information. Qualitative data were analyzed using an adapted practical thematic analysis method for health service research proposed by Saunders et al [45]. There are 3 steps of the method. The first is to read and annotate the data to understand the breadth and depth of the results. Then, the data are coded with descriptive titles that report detailed themes. After this, the low-level titles are synthesized to identify broad themes from the results. This results in 3 levels of thematic codes. An iterative procedure, conducted by multiple reviewers, was taken to ensure data saturation. NVivo (Lumivero) was used to complete thematic analysis. Quantitative data from surveys are reported but not statistically analyzed due to the small sample size and nature of the study design [46].

### Ethical Considerations

To maintain participant confidentiality, all data collected from participants were deidentified. To ensure that patient participants received the proper quality of care, patients were seen by a doctor both physically and virtually. Only the lowest risk patients were recruited for the study. Patients received a R100 grocery voucher as compensation for their time. The nurse and doctor participants were compensated at a standard hourly rate based on their qualifications for their time conducting consultations. There were no foreseen risks with participation in the study for any of the participant groups. The medical devices that were used for virtual examinations were all approved by the Federal Drug Administration and the Conformité Européenne for use in clinical diagnostics. This study was approved by the University of Cape Town Human Research Ethics Committee, reference number HREC322/2024. The relevant organizational bodies, including the Western Cape Department of Health (study number WC_202411_029), Mitchell’s Plain Community Health Centre, and SARAO SKA management, also provided approval for the study.

## Results

### Cases and Consultations

Two doctor-nurse dyads participated in the virtual clinic visits, with 1 team per site. The doctor and nurse conducting visits at site 1 had not met before data collection. The doctor and nurse at site 2 were familiar with each other and had worked together previously. A total of 12 patients were seen during the study, with 9 at site 1 and 3 at site 2. This participant recruitment size is in line with similar research studies [47]. The average patient age was 42 years, with a range of 23‐73 years. Female patients accounted for 66.6% of the population for both sites. An overview of the participants and cases is shown in Table 1. For patient participants, the presenting complaints are listed along with a brief description of the final assessment made by the doctor.

The full results of the thematic analysis are provided in Multimedia Appendix 2. The following 6 sections provide descriptions of each of the 6 themes identified and their supporting codes and evidence.

**Table 1.** Overview of patient participants and brief description of cases.

Site	Day	Age, years	Sex	Presenting complaint	Final assessment
1	1	73	Male	Pain in back and buttocks	Skin lesion
1	1	45	Female	Abdominal pain	Suspected TB^a^
1	1	44	Female	Headache and dizziness	Hypertension medication nonadherence
1	1	28	Female	Headache and cough	Flu
1	1	51	Male	Chest pain with cough	Pneumonia
1	2	43	Female	Back pain	Suspected TB
1	2	38	Male	Pain in nose and face	Gingivitis and abscessed teeth
1	2	23	Female	Abdominal pain and nausea	Bladder infection
1	2	55	Female	Body aches	Sciatica flare-up
2	3	30	Male	Raised bump on eye	Eye nodule + hypertension
2	3	32	Female	Abdominal pain	Gastritis and acid reflux
2	3	36	Female	Abdominal pain during certain activities	Potential bladder prolapse, referral to gynecology

a TB: tuberculosis.

### Characteristics of Successful Virtual Clinic Consultations

**Figure 4.**
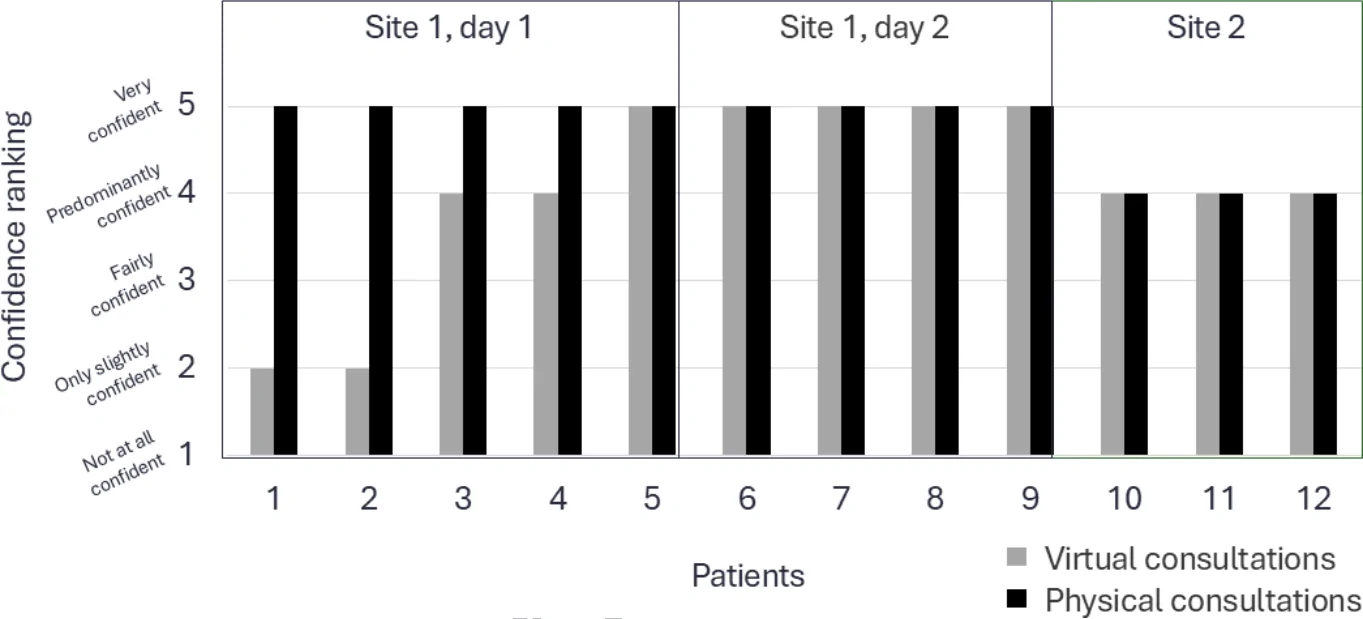
Doctor-reported Likert-scale rankings of confidence in final assessment between virtual and physical consultations for each patient.

**Figure 5.**
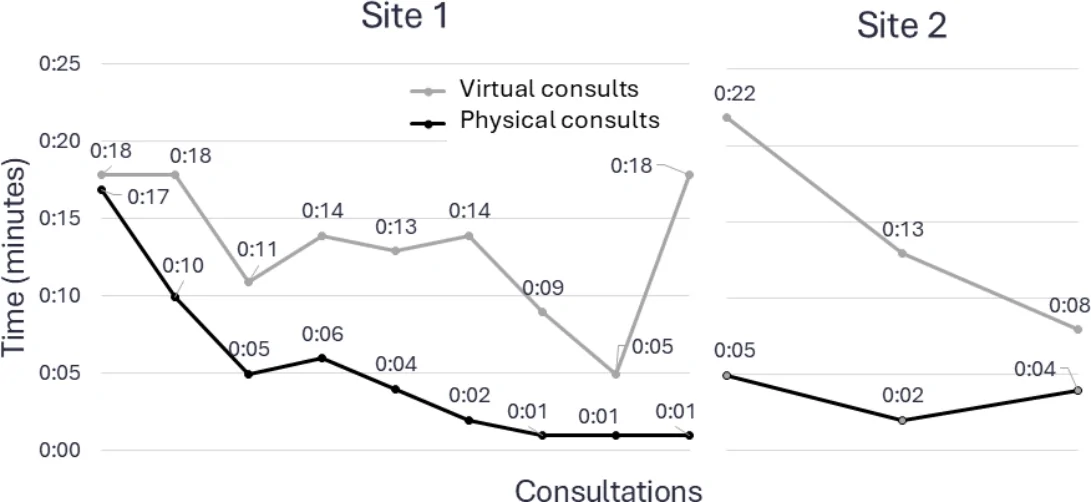
Duration of virtual and physical consultations for each site.

First, experience was an enabler of confidence and success for new users. For both doctor-nurse dyads, confidence was reported to grow over time as more consultations were conducted. Doctor’s confidence in diagnosis from virtual and physical examinations over time is reported in Figure 4, where it is demonstrated that confidence grew over time for the doctor at site 1. The confidence between consultations became equal starting at the end of day 1, and the pattern continued into day 2, demonstrating sustained confidence. In addition to doctor’s confidence, the time spent verifying the outcomes of the virtual examination during the physical examination shortened over time, as shown in Figure 5. This aligns with both doctors’ questionnaire responses, which state that as consultations progressed, they were using only the physical examination to verify the findings they had made during the virtual examination.

The nurse at site 1 had the following to say about her experience with the virtual clinic on the first day:

*It was not as bad as I thought. I really thought it was going to be hard to collect the information, [and] the patients would be uncomfortable with not knowing exactly who they're speaking to. And to patients, what they also like is they like to be touched. So I was quite uncertain about it. And then, the more we got to do it, I got more comfortable with the system*.

Both doctors mentioned the importance of being able to exclude emergency diagnoses during virtual consults. During several consultations for both doctors, emergency diagnoses could be excluded through a combination of history taking and physical examinations conducted by the nurse. For the second case on day 1, the doctor noted that she was able to exclude signs of meningitis through the nurse’s physical examination reports. The doctor at site 2 also reported that he was able to exclude emergency diagnoses for all 3 patients during the virtual consults.

The study design itself may have also contributed to building confidence in virtual diagnosis. Although not initially intended to do so, comparison between virtual and physical examinations allowed doctors to build trust in the technology and the nurse they were working with. The doctor at site 1 stated the following regarding her experience after the first day of using the system:

*Initially I was very sceptical because we rely a lot on clinical examination to make a diagnosis. Especially as South African clinicians, because we don't always have special investigations, so to touch, to feel, to smell, yeah, it does make a difference. So I was sceptical. But then as we progressed, it got easier. And then, what [the nurse] found and what I found was more or less the same thing, when we examined*.

She also had the following to say regarding the exercise of comparing virtual and physical diagnoses:

*Because like with [the patient] who needed an X-ray, which you need an in-house consultation, I'm kind of like would that actually have made a difference to my treatment in the end? Because based on what we found in the clinical examination, the X-ray just confirmed what we already decided, so I think it is beneficial just to rule out any really bad things. But, I think we did still get to the right diagnosis through the first virtual consultation*.

As consultations continued, confidence in virtual consults built among the doctors and nurses. The exercise of verification of diagnoses between virtual and physical formats is theorized to have contributed to this confidence-building, as it allowed for the establishment of trust between the nurse and the doctor. The ability to exclude emergency diagnoses is also theorized to have contributed to building confidence. The confidence demonstrated by the conclusion of the study days motivates for proof that successful virtual clinic consultations can be conducted in remote, rural, and underserved areas with the virtual clinic system.

Clear, descriptive communication between providers facilitated successful consults. An observed element of successful consultations was clear communication. This is discussed in the previous section with reference to both patients and providers, but provider-to-provider communication is also extremely important in the virtual setting. During consultations, both doctor and nurse pairs were able to conduct physical examinations over the virtual clinic platform, with the nurses providing detailed descriptions and the doctor directing the examination. Physical examinations on several areas of the body were conducted in this fashion, including the head, neck, abdomen, back, and buttocks. The doctor at site 1 noted the importance of communication in these examinations with the following:

*I think you just you need to be very good at explaining things. It helps with [the nurse] because she already asks the questions and does the things, but if you don't have someone that’s as experienced, you would just need to be able to relay that information. Like this is the particular examination that I want, [and] this is how you conduct that examination*.

These descriptions can also be important when complementing the information obtained from the medical devices. The nurse at site 1 noted during one of the examinations:

*Unclear otoscopic images made it difficult for the doctor to see clearly, and therefore ask[ed] further investigations/assessment questions*.

Both doctors noted that they liked working with a provider-to-provider virtual clinic model rather than a patient-to-provider model. The doctor at site 2 noted that having a professional nurse on the ground who has medical experience is helpful when conducting the physical examinations and could also build trust and confidence in the patient receiving the service. The site 1 team corroborated this comment with the following:

*Nurse: I'm just thinking if someone was at home and you don’t have a nurse on the other end, it would be difficult.*

*Doctor: It would be very difficult. Yeah. So what makes this flawless is having an experienced sister, because if it is a patient at home and I'm just gathering information by speaking, I can’t touch them. There is no one there that can do it. I can’t listen to the chest so I could see that guy who looks fine. Maybe just the green sputum is the only thing that would make you think infection, but it would make it difficult*.

Finally, the usefulness of this communication was highlighted by the nurse and the doctor at site 1. They stated that the ability to verify diagnoses and referrals between providers would be beneficial to building health care workers’ experience and confidence working in remote, rural, and underserved areas. When asked whether this service would be helpful to the nurse at site 1 during her previous work in the Eastern Cape, she said:

*Extremely. I remember when I was a nurse, the only thing we had was the group on WhatsApp with all of us, and then we helped each other. And that we would have one doctor that was nice enough to accept our patients and he would be like, OK, have you done this? Have you done that? And then eventually the doctor would be like, OK, you can refer. So yeah.*

The doctor at site 1 followed by stating:

*And even for junior doctors [this would be helpful]. Yeah, because you are running a hospital on your own. And you don’t have enough experience. And then you need to try and get to a specialist at the other hospital, who doesn’t necessarily have time.*

Therefore, by providing a platform for establishing communication between providers, detailed accounts of physical examinations can be shared to improve health care delivery.

### Patient Satisfaction With Virtual Care

Patient satisfaction was demonstrated when patients understood the benefits of virtual consults and were included in communication. Overall, patients lacked experience with virtual clinics but reported positive experiences after participating in a virtual consult. Results from preconsult and postconsult patient surveys are reported in Table 2. On the preconsult survey, only 1 patient participant answered yes to having previously used telemedicine services. On the postconsult surveys, patients indicated satisfaction with the system, with the majority of TeSS responses falling in the “good” or “excellent” categories. All patients reported that they would use the service again and would recommend the service to another person.

**Table 2.** Summary of the patient responses to pre- and postconsult surveys.

Survey item	Response					
	Yes, n (%)	No, n (%)	Excellent, n (%)	Good, n (%)	Poor, n (%)	Fair, n (%)
Preconsult patient survey: have you consulted a doctor or a nurse through phone or video before?	1 (8.3)	11 (91.7)	—^a^	—	—	—
Postconsult patient survey statement. How satisfied were you with:						
The voice quality of the equipment?	—	—	6 (50)	5 (41.7)	0 (0)	6 (50)
The visual quality of the equipment?	—	—	6 (50)	5 (41.7)	1 (8.3)	6 (50)
Your personal comfort in being seen using the virtual clinic system?	—	—	8 (66.7)	4 (33.3)	0 (0)	8 (66.7)
The length of time with the doctor you saw?^b^	—	—	5 (45.5)	5 (45.5)	1 (8.3)	0 (0)
The explanation of your treatment by the virtual clinic doctor?	—	—	9 (75)	3 (25)	0 (0)	0 (0)
The thoroughness, carefulness, and skillfulness of the virtual clinic doctor?	—	—	9 (75)	2 (16.7)	1 (8.3)	0 (0)
The courtesy, respect, sensitivity, and friendliness of the virtual clinic doctor?	—	—	10 (83.3)	2 (16.7)	0 (0)	0 (0)
How well the virtual clinic doctor respected your privacy?	—	—	7 (58.3)	4 (33.3)	1 (8.3)	0 (0)
How well the staff answered your questions about the equipment?^b^	—	—	5 (45.5)	5 (45.5)	1 (8.3)	0 (0)
Your overall treatment experience using the virtual clinic?	—	—	7 (58.3)	5 (41.7)	0 (0)	0 (0)
Would you recommend the virtual clinic service to another person?	12 (100)	0 (0)	—	—	—	—
Would you use the virtual clinic service again?	12 (100)	0 (0)	—	—	—	—

a Not applicable.

b Questions that were not answered by 1 respondent each.

The nurse at site 1 had the following to say about one of the patient’s demonstrated behaviors during a virtual consultation:

*At the beginning, there was the lady that came, the one who just had the headache. She was a bit unsure at the beginning. She was very edgy, but then once she realized that, actually, I’m going to see a doctor, then I think she started relaxing*.

**Figure 6.**
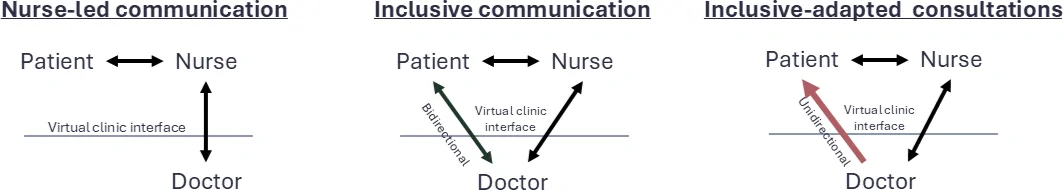
Communication styles observed during virtual consultations.

An observation during consultations was that the model of communication between the doctor, nurse, and patient was not always consistent. The observed models of communication are demonstrated in Figure 6. The optimal mode of communication was when the patient, nurse, and doctor all spoke directly to one another, labeled as inclusive communication. This also involved using a video view where both the patient and the nurse were included. This was important for the doctor to see nonverbal communications, such as body language or shaking of the head “yes” or “no” to answer questions. In one case, the doctor was able to determine that the patient was nasally congested by speaking directly to the patient and hearing their voice. However, in some cases, inclusive communication was not used. Particularly, these were in earlier consultations for site 1, but in some cases, this style of communication was also not feasible. Two other models of communication were thus observed, and these were labeled nurse-led and inclusive-adapted. In nurse-led communication, the nurse fully facilitated communication between the doctor and the patient. This was necessary during consultation 1 on the first day, when the patient was hard of hearing and the doctor’s comments had to be repeated to him by the nurse. In inclusive-adapted communication, the doctor spoke directly to the patient, but the patient would respond through the nurse. Whether the patient intended to speak to the doctor directly was difficult to assess; in some cases, the patient was soft-spoken, and in other cases, their body language indicated that they were speaking to the nurse. In both the nurse-led and inclusive-adapted models, there was increased repetition of information that could have contributed to longer consultation durations. However, these styles of communication may be more comfortable for certain patients and thus should not be discounted. Rather, nurses and doctors should be informed about these styles of communication and be directed to make a conscientious choice that balances patient comfort and communication efficiency.

Additionally, patients who participated in the study may have received higher-quality care than they would have received in the traditional clinic setting. This was mentioned as particularly true for the green-triaged patients at site 1, who often face extremely long wait times as the day progresses, when more emergency cases take priority. In fact, 2 patients were recruited for the study on day 1, approximately 1 hour after the clinic opened. Neither patient met the inclusion criteria and was thus not included in the study. At the conclusion of the study day on day 1, approximately 5 hours after the nonincluded patients had entered the clinic, these patients were still in the queue and had not yet been seen. The long wait times are corroborated by the doctor and the nurse at site 1, who have previous experience working in other public clinics across the country.

*Doctor: [The virtual clinic] actually makes a big difference, because we have a lot of clinics [that] don’t have doctors and then that patient gets referred to a facility with a doctor. And it takes them weeks or months to get a date to see the doctor*.

*Nurse: It’s actually hard to get a date, especially in the rural areas. I'm not looking just at the Western Cape, but thinking Eastern Cape*.

While the quality-of-care impact is stark for the patients recruited in this study because they were green, or low priority, patients, it is recognized that this may not be the case for all virtual clinic implementations. However, what can be acknowledged is that patients were satisfied to receive care from a real doctor and nurse, with a physical examination component, and that these elements could be provided by the virtual clinic service.

### Barriers to Virtual Clinic Care Delivery

Barriers caused by infrastructure and the implementation environment were able to be addressed adequately during the study. They did, however, threaten the success of the virtual consultations. Therefore, proper consideration should be given to these barriers to ensure that they have been addressed adequately.

On day 1 of the site 1 implementation, several technical issues were encountered. As this was the first implementation of the virtual clinic in a real-world setting, this was not surprising. The major challenge was establishing a quality network connection. The doctor was connected to the virtual clinic app using a tablet, which was receiving internet connection from a mobile phone hotspot that was connected to a local mobile network provider. The nurse’s computer was connected directly to the mobile network, using a USB SIM modem. Throughout day 1, there were intermittent disconnections along with video and audio lag, which disrupted virtual consults. The mobile phone hotspot was suspected to be contributing to the poor connection, and various SIM cards, phones, and settings were adjusted throughout the day to try to improve this. It was also attempted to use USB tethering to hotspot the mobile network, and with this, the network quality improved. However, this caused the battery of the tablet to drain quickly, and the tablet died, interrupting a virtual consultation. With these challenges, virtual consults could still take place, but the duration and confidence associated with these consults were low. These challenges were highlighted in a written response by the doctor referencing a consult with network issues:

*Video lag. Network issues. Online consultation tends to take long due to lag issues (network)*.

The nurse also noted the potential risks with loss of network connection during one of the consultations:

*Only 1 difficulty was encountered, network glitching. Resulted in the doctor repeating suggestion/questions, or possibly missing some information from my side to the doctor or from the doctor to my side*.

There were several actions taken between day 1 and day 2 at site 1 to improve the quality of the virtual clinic consultations, and these were carried over into the site 2 study day. The laptop webcam was substituted by a high-quality HD1080p camera, and a USB SIM modem was used to provide mobile network to a laptop on the doctor’s side, instead of a tablet. The pair of headphones that the doctor had been using was adjusted to provide better sound quality. These changes had a drastic impact on the success of virtual consultations. There were no observed or mentioned network challenges on day 2 at site 1, despite using the same network providers and the same location. This indicates that the network challenges experienced on the first day were due to the hardware used and not due to the service provider.

Some contextual factors of the clinic environment caused brief challenges in communication, particularly due to the background noise. At site 1, there were intercom announcements that interrupted communication, and at site 2, there were construction noises and a heater which caused background noise. However, the doctor at site 2 noted that the background noise was present but not disturbing when using the headphones.

Another environmental factor that affected virtual consultations was the arrangement of the examination space. At site 1, the examination bed had to be positioned approximately 4 meters from the laptop and camera, while at site 2, the examination bed could be positioned only 1 meter away. The arrangement at site 1 caused challenges during the demonstration of physical examinations that were not present during site 2 consultations. The nurse at site 1 reported:

*Since the laptop is situated on the desk and the bed is further away, when doing an abdominal exam, it would be nice if the video camera would be closer so the doctor can see what areas are being palpated and what the abdomen looks like, direct where to palpate, and what kind of palpations/assessments to do*.

It is important to note that if these environmental factors are not considered, it threatens the ability of the system to streamline existing clinical workflows. The doctor at site 1 mentions this, saying:

*So if I was in a busy setting, kind of having to communicate via another person to speak to the patient, there is time that that takes, whereas I wouldn't even have sat and asked as many questions as I usually do. So you would need to have a flawless system where there’s no lags in between all of the [consults]. Yeah, it would not be as relaxed as it is now. Because if I had to see 30 patients in this time, [or] five who took so long?*

As such, pilot studies such as this are extremely important in identifying these concerns and mitigating them through future design and recommendations. Another barrier encountered during virtual clinic consultations was that medical device data were not always suitable for conveying certain types of health information. During consultations, the medical devices were used with the virtual clinic platform to provide real-time health data. An overview of how the medical devices were used for each consult is provided in Table 3. The vital signs monitor was not included in the table, as no consult required electrocardiogram usage. During initial consults at each site, the vital signs monitor was used for vital signs examination. However, at site 1, the triaging report data were used from consult 3 onward to capture patients’ vital signs. At site 2, the nurse preferred to use the clinic’s devices to take measurements, potentially due to the familiarity that they provided.

**Table 3.** Medical device usage during virtual consults.

Site	Day	Medical device usage (yes/no) and description of usage	
		Medical camera	Stethoscope
1	1	No	No
1	1	Yes: pupil reactivity to light	No
1	1	Yes: pupil reactivity to light, inside ear, and throat	No
1	1	Yes: inside throat	Yes: chest examination
1	1	No	Yes: chest examination
1	2	No	Yes: chest examination
1	2	Yes: inside mouth and throat	No
1	2	No	No
1	2	No	No
2	3	Yes: eyelid examination	Yes: chest examination
2	3	No	Yes: abdominal examination
2	3	No	No

The medical devices were not always able to properly convey what the nurse saw or heard on the ground. The stethoscope was reported to provide variable sound quality. When comparing the performance of the digital stethoscope with her own stethoscope, the doctor at site 1 noted:

*Digital stethoscope note: audio very loud can over-amplify sound which could lead to overinterpretation of pathology. Low frequencies are very loud, high frequencies not audible*.

For the first patient at site 2, the doctor could not hear the breath or heart sounds from the digital stethoscope, as can be seen from the following note:

*Stethoscope sound quality was not sufficient to assess heart sounds*.

However, the sounds could be heard for the subsequent 2 patients at site 2, and the doctor reported high sound quality for these patients. The doctor at site 2 determined that this could be due to the faintness of the breath and heart sounds in the first patient, which the nurse could hear but he could not. Therefore, while the stethoscope demonstrated satisfactory performance for some patients, this was not consistent between patients. Furthermore, the examination camera was more useful in sharing dermatological images rather than otoscopic images. The nurse at site 1 provided the following comment:

*During this consultation it was a bit challenging to demonstrate clearly using the otoscope, to show the mouth clearly. For the light (otoscope) I tried removing the black head and focuser but the light wasn’t able to clearly show the focused part of the mouth/throat and show the gingivitis/swelling and infection in the gums and teeth. After removing the black head for the clear head and focuser, it still wasn’t easy to see the mouth/gums/teeth. It seems the colour of the light (light blue light) doesn’t work well, we tried using the light from the ward’s otoscope and it was more yellow/orange and the viewing/imaging was much better in that light*.

So, to alleviate the challenges from the medical camera, the device was used in combination with the light provided from the clinic otoscope. In addition, it was mentioned several times that the medical camera could not show a clear view of the tympanic membrane in the ear. This was a similar comment received during usability testing, which motivated a redesign of the otoscope to allow for a narrower tip to navigate the ear canal. However, this redesign was not able to change the design of the device itself, which may need to be adjusted to improve this capability.

Although the devices used were Federal Drug Administration– and Conformité Européenne–approved, they did not end up providing the quality necessary during all examinations. The evidence provided supports user-guided feedback on how the devices may be improved. These conclusions also motivate further development of open medical devices that are not tied to proprietary software, as the market options for these types of devices are limited. Development and improvement of these devices for use in systems such as the developed virtual clinic will be advantageous to providing better quality virtual care.

Finally, there were limitations of the virtual clinic system in providing primary health-level services. While most consults conducted using the virtual clinic were successful, there were some limitations of the system that should be mentioned. Some challenges simply cannot be addressed, given the current technical capabilities of virtual consultation. For example, the nurse at site 1 highlights one piece of information she struggled to share with the doctor during a consult:

*The warm of the patient’s hands, that had to be described extensively*.

At the current state, it would not be feasible to add a device that can convey this information with certainty; however, there could be additional training or research in description or examination that could assist with conveying the necessary information to the doctor. Ultimately, it was not within the scope of this study to determine what cases are not appropriate for virtual care and which are best suited, but this is recommended for further research.

## Discussion

### Principal Findings

The results from this study inform recommendations for digital health systems for remote, rural, and underserved areas. These recommendations are summarized in Table 4. There were still some areas for improvement in the system design, as demonstrated by clinical implementation feedback, but most of the user feedback centered around implementation feedback rather than system design feedback. This transition demonstrates how UCD-based iteration proved effective in taking user feedback to develop an improved virtual clinic system that addressed user needs [28].

**Table 4.** Summary of virtual clinic recommendations.

Result	Supporting evidence	Recommendation
Experience is an enabler of confidence and success for new users.	As consults progressed, doctors reported less reliance on the follow-up physical examinations to diagnose. Physical consult time reduced as consults progressed. Doctors’ confidence in virtual diagnosis increased as consults progressed.	Nurse and doctor users should participate in training and hands-on practice with the system.
Patients’ satisfaction is demonstrated when patients understand the benefits of virtual consults and are included in communication.	Patients reported high satisfaction with the virtual clinic system postconsultation. Wait times were reduced for patient participants in comparison with other patients in the clinics. Three communication styles emerged, which had different effects on patient involvement during the virtual consult.	Nurse and doctor users should be educated on the different communication styles for virtual consults and apply them based on the patient context. Patients should be educated on the potential positive impacts of virtual consults to encourage adoption.
Clear, descriptive communication between providers facilitates successful consults.	Nurses used verbal descriptions to convey the patient’s condition to the doctor during virtual consultations. Doctors could confidently diagnose patients based on verbal description supplemented with medical device data.	Nurse and doctor users should be trained together on using descriptive verbal communication during virtual consults. Nurse and doctor users should practice virtual and physical consults together to build trust in one another’s skills.
Barriers caused by infrastructure and the implementation environment can be addressed adequately but must not be ignored; otherwise, success could be threatened.	Changing the network access hardware for the virtual clinic app correlated with better quality of service and an increase in doctors’ confidence in virtual diagnosis. Nurse and doctor users commented positively on the higher-quality camera implemented on the second day of study.	High-quality periphery devices (cameras, headphones, and network access devices) should be used with the virtual clinic app to enable virtual consult success. The physical clinic setup should support virtual consults, including positioning the examination bed and the patient near the camera and microphone.
Medical device data may not be suitable for conveying certain types of health information.	The stethoscope device did not convey soft or shallow breath and heart sounds adequately for the remote doctor to hear. The medical camera could not show mouth, teeth, and ear images adequately for the remote doctor to see.	Existing medical devices should be improved based on the user’s feedback provided in this study. There should be more open medical devices for open software apps, such as the virtual clinic. Nurses should be trained on how to supplement medical device data with verbal descriptions.
There are limitations of the virtual clinic system in providing primary health-level services.	The virtual clinic system could not convey data regarding the warmth of a patient’s hands, which the nurse deemed important for diagnosis.	The constraints of the virtual clinic system should be further defined based on additional studies. The virtual clinic system should consider the health data requirements of the implementation context and the feasibility (cost and market availability) of the technology used to convey these health data.

The user feedback provided during clinical implementation aligns with the Capabilities, Opportunities, Motivation, and Behavior (COM-B) framework for behavioral change [48]. COM-B states that users need the capacity to engage, a conducive sociophysical environment, and the drive to perform a behavior. In this context, capability refers to the user’s ability to interact with the system, which is supported by help desk materials and exposure to the system. Opportunities can involve the infrastructure used to support the implementation. Motivation included the intrinsic desire of the health care worker to provide proper care to their patients [49], which was enabled by the virtual clinic system. This result has been reported in the development of other digital health solutions that used UCD principles [50,51]. Doctor and nurse participants highlighted the need for a solution in the public health care system and expressed a greater tendency for behavior change after they felt more capable of using the system. Working in teams was an enabler of success for both users, as trust was built and effective communication styles were established. This aligned with similar findings of virtual clinic implementations [52]. The threats of the physical environment and technology that were experienced, such as the network issues on day 1 of implementation, did have a negative effect on the provider’s experience with the system, aligning with the opportunities aspect of COM-B and the consensus in literature that technological functionality has an effect on provider telemedicine satisfaction [53]. Additionally, there was a demonstrated improvement from the previous results of usability testing [32], where users indicated that they would like more training with the system. This aligns with the capability aspect of the COM-B framework, as users were provided an opportunity to build their confidence with the virtual clinic system before consulting with patients [54]. This included hardware and software components. The help desk subsystem, including the hands-on training that was incorporated, provided for this aspect of motivation. This design also supported potential threats identified, such as a lack of generational digital literacy [55]. Motivation was still clearly existent, as the providers also understood the needs of the health care system and the benefits that the virtual clinic system could provide. Therefore, when the technological challenges were addressed, all factors of the COM-B framework were addressed, and participants had a greater likelihood of wanting to implement the virtual clinic system. At site 2, these challenges had been addressed, and therefore quicker uptake of the virtual clinic system was demonstrated.

The results of this evaluation serve as a proof of concept that the system can work in real-world environments and motivate for further research and implementation based on these recommendations. Not only does this information provide insights into the virtual clinic, but it also provides insights into the landscape and readiness of South Africa for digital health implementations. The information, such as patient satisfaction with virtual clinic services, provider opinions toward virtual clinic consultations, quality of service of various African and South African networks, and hardware considerations for better design, motivates for implementation of virtual clinic and digital health solutions in these areas.

### Conclusions

The results of this study support the acceptability and feasibility of the developed virtual clinic system in a real-world setting. Furthermore, the results of this study provide evidence of virtual clinics in remote, rural, and underserved areas of South Africa. This research highlighted that provider experience and confidence with using the digital health system was an enabler of successful virtual clinic consultations. Additionally, clear communication between the health care worker in the remote, rural, or underserved area and the remotely located doctor resulted in quicker consultations. Patient feedback emphasized that when providers directly communicate with patients during virtual consultations, and when the patient understands the benefits of using virtual clinic consultations, patients have improved experience. Finally, barriers that are discussed in similar implementations, including limited infrastructure [11-13] and contextual challenges [14,15], were also observed during this study. However, these contextual challenges could be overcome with appropriate design methodologies such as UCD. This included selection of medical devices that align with the needs of the context and the limitations of the environment.

The current evidence of interventions in remote, rural, and underserved areas is limited; therefore, these results contribute not only to the development of this system but also to the development of other digital health solutions. The nature of the feedback and observations provides insights into the motivation and behaviors of doctors, nurses, and patients using virtual clinic services in low-resource South African contexts. The recommendations from the Results section provide a framework of how other digital health developers might consider implementation in these contexts, aligning with the monitoring and evaluation phase of the World Health Organization Telemedicine Implementation Guidelines [16].

There were some methodological and contextual limitations of this study. There was a lack of additional reviewers and coders used during thematic analysis. While this is important to reduce bias, other measures, such as reporting direct quotes and performing iterative thematic analysis, were introduced to reduce bias. Considering the patient population recruited, there may have been bias in patient satisfaction with the system, particularly at site 1. This is because patients who were recruited were green-triaged patients, who are the lowest priority patients at the clinic. Often, these patients face the longest wait times as the other, higher priority patients take precedence over them. Some green-triaged patients do not see a doctor at all when entering the clinic, particularly on a weekend when clinic staff is limited. They may also be limited to seeing a nurse only, and not a doctor at the clinic, depending on their complaint. Their participation in the study allowed them to see a doctor almost immediately after being triaged at the clinic. As such, they may have indicated satisfaction due to the speed and quality of care they were provided by their participation in the study. Given this, further validation of patient satisfaction results during long-term virtual clinic implementation is needed. Finally, it is suggested that additional studies incorporate a direct comparison between diagnosis during virtual and diagnosis during physical examinations. This allows for a better comparison between the 2 interventions.

## Supplementary material

10.2196/84272 Multimedia Appendix 1 Study questionnaires.

10.2196/84272 Multimedia Appendix 2 Thematic analysis results.

## Abbreviations

COM-B: Capabilities, Opportunities, Motivation, and Behavior
SARAO: South African Radio Astronomy Observatory
SKA: Square Kilometre Array
TeSS: Telehealth Satisfaction Survey
UCD: user-centered design
