# Supplementary material for: User-Centered Virtual Clinic Services for Remote, Rural, and Underserved Sub-Saharan Africa: Development and Usability Study
Source: JMIR Hum Factors. 2026 Sep 1;13:e84272. doi: 10.2196/84272 (PMC13533315; doi:10.2196/84272)
Supplement: Supplementary file 1 [file humanfactors-v13-e84272-s001.docx]

# Multimedia Appendix 1

# Study Questionnaires

### Post-Virtual-Consult Survey for Doctors

1. What additional information will you aim to get from the physical consultation?
2. Please mention briefly any difficulties you experienced, if any.
3. Please rank your confidence in your current final assessment:

| **Not at all confident** | **Only slightly confident** | **Fairly Confident** | **Predominantly Confident** | **Very**  **Confident** |
| --- | --- | --- | --- | --- |
| 1 | 2 | 3 | 4 | 5 |

### Post-Physical-Consult Survey for Doctors

4. Referencing question 1 [of the post-virtual-consult survey for doctors], were you able to get this information from the physical exam? Y/N
5. If no to the previous question, explain.
6. Please rank your confidence in your current final assessment:

| **Not at all confident** | **Only slightly confident** | **Fairly Confident** | **Predominantly Confident** | **Very**  **Confident** |
| --- | --- | --- | --- | --- |
| 1 | 2 | 3 | 4 | 5 |

### Post-Consult Survey for Nurses

1. What patient information (if any) were you not able to properly demonstrate during the virtual consultation?
2. Please describe any difficulties you experienced during this consultation, if any.

### Pre-Consult Survey for Patients

1. Age:
2. Sex: M/F/Prefer not to disclose
3. Have you consulted a doctor or nurse through phone or video before? Y/N

### Post-Consult Survey for Patients

| *How satisfied were you with:* | *Poor* | *Fair* | *Good* | *Excellent* |
| --- | --- | --- | --- | --- |
| 1. The voice quality of the equipment? | 1 | 2 | 3 | 4 |
| 2. The visual quality of the equipment? | 1 | 2 | 3 | 4 |
| 3. Your personal comfort in being seen using the virtual clinic system? | 1 | 2 | 3 | 4 |
| 4. The length of time with the doctor you saw? | 1 | 2 | 3 | 4 |
| 5. The explanation of your treatment by the virtual clinic doctor? | 1 | 2 | 3 | 4 |
| 6. The thoroughness, carefulness and skilfulness of the virtual clinic doctor? | 1 | 2 | 3 | 4 |
| 7. The courtesy, respect, sensitivity and friendliness of the virtual clinic doctor? | 1 | 2 | 3 | 4 |
| 8. How well the virtual clinic doctor respected your privacy? | 1 | 2 | 3 | 4 |
| 9. How well the staff answered your questions about the equipment? | 1 | 2 | 3 | 4 |
| 10. Your overall treatment experience using the virtual clinic? | 1 | 2 | 3 | 4 |

11. Would you recommend the virtual clinic service to another person? Y/N
12. Would you use the virtual clinic service again? Y/N
13. Please provide any further comments about your experience today.
