# Supplementary material for: User-Centered Virtual Clinic Services for Remote, Rural, and Underserved Sub-Saharan Africa: Development and Usability Study
Source: JMIR Hum Factors. 2026 Sep 1;13:e84272. doi: 10.2196/84272 (PMC13533315; doi:10.2196/84272)
Supplement: Supplementary file 2 [file humanfactors-v13-e84272-s002.docx]

## Multimedia Appendix 2

## Thematic Analysis Results

*Participant Identifiers Key:*

D = doctor; N = nurse; P = patient; number following D or N indicates Site. Number following P indicates consultation number out of 12.

*Code Key:*

1. Theme
   a. Code
      i. Supporting evidence
1. Experience is an enabler of confidence and success for new users.
   a. Successful consultations grew over time and required shorter physical consults for verification.
      i. N1: “It was not as bad as I thought. I really thought it was going to be hard to collect the information, [and] the patients would be uncomfortable with not knowing exactly who they're speaking to. And to patients, what they also like is they like to be touched. So I was quite uncertain about it. And then, the Dre we got to do it, I got more comfortable with the system.”
      ii. D2 mentioned feeling much more confident on this second exam, and being able to direct the nurse on how to examine the patient.
      iii. D2 stated after the first consult that he could have guided the nurse further during the palpation to get more information.
   b. Comparison between virtual and physical exams allowed doctors to build trust in the technology and the nurse on the ground.
      i. D1: “Initially I was very sceptical because we rely a lot on clinical examination to make a diagnosis. Especially as South African clinicians, because we don't always have special investigations, so to touch, to feel, to smell, yeah, it does make a difference. So I was sceptical. But then as we progressed, it got easier. And then, what [the nurse] found and what I found was more or less the same thing, when we examined.”
      ii. D1: “Because like with [the patient] who needed an X-ray, which you need an in-house consultation, I'm kind of like would that actually have made a difference to my treatment in the end? Because based on what we found in the clinical examination, the X-ray just confirmed what we already decided, so I think it is beneficial just to rule out any really bad things. But, I think we did still get to the right diagnosis through the first virtual consultation.”
   c. Exclusion of emergency cases was important to establish, and was possible through the virtual examination.
      i. D2 mentioned being able to exclude emergency cases during this assessment
2. Patient satisfaction is demonstrated when patients understand the benefits of virtual consults are and are included in communication.
   a. Patients lacked experience with virtual clinics, but reported positive experiences after their virtual consults.
      i. N1: “So I think you saw [P04], yeah, at the beginning. Yeah. At the beginning, there was the lady that came, the one who just had the headache. She was a bit unsure at the beginning. She was very edgy, but then once she realized that, actually, I'm going to see a doctor, then I think she started relaxing. Yeah. So at the beginning, I think [the patients] were like, “Is this a waste of my time? Should I go back to the queue?” because you know, but then once they realize that, “Oh, actually there's a nurse and there's a doctor. Oh, well, then I'm [going to] get care.” So I think that will be possible, and then feel that we're doing like some of the things that inside [the clinic], they were not going to have access to.”
   b. Successful consults included the patient directly in the consultation discussion and camera view.
      i. P3152 Observation: Call dialogue occurs between nurse and doctor only. The patient is not in the video unless necessary. The doctor and patient can hear one another in most instances. Nurse repeats some comments from patient to doctor. Doctor cannot see patient body language – patient has shook her head “no” in response to a question, and nurse must verbally state the question response.
      ii. P3153 Observation: Doctor noticed that the patient was congested from their voice. The camera view during this consultation included the nurse and patient simultaneously.
      iii. P3154 Observation: Patient physically demonstrated where the pain of the headache was over the video.
   c. Patients received a higher quality of care through the virtual clinic.
      i. D1: “[The virtual clinic] actually makes a big difference, because we have a lot of clinics [that] don’t have doctors and then that patient gets referred to a facility with a doctor. And it takes them weeks or months to get a date to see the doctor.”

N1: It's actually hard to get a date, especially in the rural areas. I'm not looking just at the Western Cape, but thinking Eastern Cape.

ii. D1: “Yeah, yeah, because if the [patient] were to go inside, they wouldn't have gotten extra time. [But here] I was just like we have time. We can see them. Even [P05] wouldn't have gotten an X-ray.

3. Clear, descriptive communication between providers facilitates successful consults.
   a. Physical exams that are conducted by the nurse and described to the doctor can provide suitable detail for diagnosis.
      i. D1: “I think you just you need to be very good at explaining things. It helps with [the nurse] because she already asks the questions and does the things, but if you don't have someone that's as experienced, you would just need to be able to relay that information. Like this is the particular examination that I want [and] this is how you conduct that examination.”
      ii. There was a physical exam performed by the nurse to palpate the kidney (while standing). Another exam was performed on the abdomen, while the patient laid down. The doctor had the patient indicate when she felt pain as the nurse moved her hand across abdomen, and the doctor watched.
   b. Effective description of exam findings can supplement image and audio data from medical devices.
      i. Doctor and nurse mentioned not being able to get a clear view of the tympanic membrane with the otoscope, as it was too dark. The nurse instead used the clinic otoscope and described what she saw.
      ii. N1: “Unclear otoscopic images made it difficult for the doctor to see clearly, and therefore ask[ed] further investigations/assessment questions.”
   c. Doctors are most comfortable with the provider-to-provider model rather than the patient-to-provider model, because clinical knowledge and experience on the ground is still required.
      i. N1: I'm just thinking if someone was at home and you don’t have a nurse on the other end, it would be difficult.

D1: “It would be very difficult. Yeah. So what makes this flawless is having an experienced sister, because if it is a patient at home and I'm just gathering information by speaking, I can't touch them. There is no one there that can do it. I can't listen to the chest so I could see that guy who looks fine. Maybe just the green sputum is the only thing that would make you think infection, but it would make it difficult.”

ii. D2 said that he would not be able to direct someone to use an ultrasound for examination who did not already know how to perform ultrasound.
d. Advice between providers supports healthcare worker capability in areas without doctors.
   i. N1: “As a community nurse practitioner, we would refer a patient if, for example, their blood [tests] or, if they came to see you as a nurse and then they come back again the second time and then you have their bloods right in front of [you] – like yes, these are the results, for example the CD4 count is less than 12, is positive then you refer to a doctor to start for conicals, things like that. So in the primary healthcare sector, ideally with access to the HECTIS, the tracking portal, OK, well then that that I think it would be fine.”
   ii. N1 [in response to, would this be a helpful service for you?]: “Extremely. I remember when I was a nurse, the only thing we had was the group on WhatsApp with all of us, and then we helped each other. And that we would have one doctor that was nice enough to accept our patients and he would be like, OK, have you done this? Have you done that? And then eventually the doctor would be like, OK, you can refer. So yeah.”

D1: “And even for junior doctors [this would be helpful]. Yeah, because you are running a hospital on your own. And you don't have enough experience. And then you need to try and get to a specialist at the other hospital, who doesn't necessarily have time.”

4. Barriers caused by infrastructure and the implementation environment can be addressed adequately, but must not be ignored otherwise success could be threatened.
   a. Investment in high quality periphery devices, including webcams, mics, and headphones, enabled better health data sharing and limited background interruptions.
      i. D1: “And the abdominal swelling as well, which, like, maybe you would be able to see if there was like better signal and an HD camera. Yeah, it would have been better.”
      ii. Sound and echo from the hallway can be heard during the consult.
      iii. N1: “N1: “The new camera [HD] camera has much better quality.”
      iv. Consultation dialogue paused when announcements were being made over the intercom at the clinic.
   b. Low quality network devices can cause throttling that deteriorates call quality, even if the network available is sufficient for facilitating calls.
      i. N1: “Only 1 difficulty was encountered, network glitching. Resulted in the doctor repeating suggestion/questions, or possibly missing some information from my side to the doctor or from the doctor to my side.”
      ii. D1: ““Video lag. Network issues. Online consultation tends to take long due to lag issues (network).”
      iii. D1: “So if I was in a busy setting, kind of having to communicate via another person to speak to the patient, there is time that that takes, whereas I wouldn't even have sat and asked as many questions as I usually do. So you would need to have a flawless system where there's no lags in between all of the [consults]. Yeah, it would not be as relaxed as it is now. Because if I had to see 3 patients in this time, [or] five who took so long?”
   c. The exam bed should be arranged close to the camera to facilitate clear video during physical exams.
      i. N1: “Since the laptop is situated on the desk and the bed is further away, when doing an abdominal exam, it would be nice if the video camera would be closer so the doctor can see what areas are being palpated and what the abdomen looks like, direct where to palpate, and what kind of palpations/assessments to do.”
5. Medical device data may not be suitable for conveying certain types of health information.
   a. The stethoscope was not effective for patients with soft auscultation sounds (due to larger body mass or shallow breath), despite performance that was stated as equal or greater than traditional stethoscopes for other patients.
      i. N1: “Yeah, I really think so. With regards to the stethoscope issue, because from my side I heard a slight crackle. It was very, very, like very low. But then what you heard on the other side was much”

D1: “Yeah. Was very pronounced, so I came in. I was. Like what is that thing in this, and just like no, there's something there. But it doesn't sound like that. And I listen. I was like oh, now I see what you're [hearing].”

N1: “But it's very low.”

ii. D1: “Digital stethoscope note: audio very loud can over-amplify sound which could lead to overinterpretation of pathology. Low frequencies are very loud, high frequencies not audible.”
iii. D2: “Stethoscope sound quality was not sufficient to assess heart sounds.”
iv. D2: “Doctor determined that the stethoscope is not good for hearing very soft sounds, but better than a traditional stethoscope for louder sounds.”
b. The exam camera was Dre successful at sharing dermascopy and pupil examinations than for ear, nose, and throat exams (despite its redesign).
   i. N1: “The ear exam was not clear. The throat exam was not clear. The otoscope images/video was not clearly demonstrating images to the doctor.”
   ii. N1: “[Could not demonstrate] if there were any swelling/distension on the sinus areas. I had to be as descriptive as possible. There was no sinus area swelling, but I think it would be difficult to show any distension/swelling in the sinus area with the imaging technology.”
   iii. N1: “During this consultation it was a bit challenging to demonstrate clearly using the otoscope, to show the mouth clearly. For the light (otoscope) I tried removing the black head and focuser but the light wasn’t able to clearly show the focused part of the mouth/throat and show the gingivitis/swelling and infection in the gums and teeth. After removing the black head for the clear head and focuser, it still wasn’t easy to see the mouth/gums/teeth. It seems the colour of the light (light blue light) doesn’t work well, we tried using the light from the ward’s otoscope and it was Dre yellow/orange and the viewing/imaging was much better in that light.”
   iv. N1: “I could see the ears, the nose and throat. But [D1] had a little bit of difficulty. And I could see on the screen that there's no way she can see it.”

6. There are limitations of the virtual clinic system in providing primary health-level services.
   a. Proposed changes are recommended before further system usage, such as workflow streamlining and bug fixes.
      i. D1: “So if I was in a busy setting, kind of having to communicate via another person to speak to the patient, there is time that that takes, whereas I wouldn't even have sat and asked as many questions as I usually do. I would just look like a spot that nurses, OK, write a script, next, in which you would like quickly go through all the systems. Just by spotting the. Listen and you know, listening here, feeling there. So you would need to have a flawless system where there's no lags in between all of the [consults]. Yeah, it would not be as relaxed as it is now. Because if I had to see 3 patients in this time, then five who took so long.”
   b. Future changes include addition of non-essential features and features that are not technically feasible at this time.
      i. N1: “The warm of the patient’s hands, that had to be described extensively.”
   c. Protocols based on further research should be developed to determine when virtual consults are appropriate.
